# A medical chart audit to assess endocrinologist perceptions of the burden of endogenous Cushing’s syndrome

**DOI:** 10.1007/s11102-023-01371-y

**Published:** 2024-01-08

**Authors:** Gabrielle Page-Wilson, Bhagyashree Oak, Abigail Silber, James Meyer, Matthew O’Hara, Eliza B. Geer

**Affiliations:** 1 Division of Endocrinology, Columbia University Irving Medical Center, New York, NY USA; 2 Trinity Life Sciences, Waltham, MA USA; 3 Xeris Pharmaceuticals, Inc, Chicago, IL USA; 4 Multidisciplinary Pituitary and Skull Base Tumor Center, Memorial Sloan Kettering Cancer Center, 1275 York Avenue, New York, NY 10065 USA

**Keywords:** Cushing’s syndrome, Cushing’s disease, Pituitary tumor, Hypercortisolism

## Abstract

**Purpose:**

This study was undertaken to assess the unmet needs within the endogenous Cushing’s syndrome (CS) care paradigm from the endocrinologist’s perspective, including data abstracted from patient charts. The study evaluated endocrinologists’ perceptions on burden of illness and treatment rationale along with the long-term clinical burden of CS, tolerability of CS treatments, and healthcare resource utilization for CS.

**Methods:**

Retrospective medical chart data from treated patients with a confirmed diagnosis of CS was abstracted using a cross-sectional survey to collect data from qualified endocrinologists. The survey included a case report form to capture patient medical chart data and a web-enabled questionnaire to capture practitioner-level data pertaining to endocrinologists’ perceptions of disease burden, CS treatments, and treatment attributes.

**Results:**

Sixty-nine endocrinologists abstracted data from 273 unique medical charts of patients with CS. Mean patient age was 46.5 ± 13.4 years, with a 60:40 (female:male) gender split. The mean duration of endogenous CS amongst patients was 4.1 years. Chart data indicated that patients experienced a high burden of comorbidities and symptoms, including fatigue, weight gain, and muscle weakness despite multi-modal treatment. When evaluating treatments for CS, endocrinologists rated improvement in health-related quality of life (HRQoL) as the most important treatment attribute (mean score = 7.8; on a scale of 1 = Not at all important to 9 = Extremely important). Surgical intervention was the modality endocrinologists were most satisfied with, but they agreed that there was a significant unmet treatment need for patients with CS.

**Conclusion:**

Endocrinologists recognized that patients with CS suffered from a debilitating condition with a high symptomatic and HRQoL burden and reported that improvement in HRQoL was the key treatment attribute influencing their treatment choices. This study highlights unmet needs for patients with CS. Patients with CS have a high rate of morbidity and comorbidity, even after treatment.

## Introduction

Endogenous Cushing’s syndrome (CS) is a rare, debilitating disorder caused by chronic overproduction of cortisol [1–3]. CS has an estimated incidence of 0.7 to 2.4 cases per million per year, with a majority of cases (~ 70%) occurring in women [1, 4, 5]. Active CS is characterized by a variety of signs and symptoms, including muscle weakness, obesity, depression, menstrual changes, facial redness, decreased libido, hirsutism, acne, ecchymoses, hypertension, diabetes, and neurocognitive deficits [6]. Because of the diverse constellation of associated symptoms, many of which are common in the general population, CS can be challenging to diagnose and patients often seek input from multiple specialists (i.e., orthopedists, rheumatologists, gynecologists, and endocrinologists) prior to receiving a correct diagnosis [6].

Current treatment options for CS include surgery as the first line of treatment, followed by pharmacotherapies as the second line option and radiation therapy, among other treatments, as a potential third line option. Pharmacotherapies include steroidogenesis inhibitors (e.g., ketoconazole, levoketoconazole, metyrapone, osilodrostat, mitotane), glucocorticoid receptor antagonists (e.g., mifepristone), and medications that inhibit tumoral ACTH secretion (e.g., pasireotide, cabergoline) [6–10]. These pharmacotherapies can be administered as monotherapy or in combination.

The impact of CS on overall health-related quality of life (HRQoL) has been previously described [11]. However, studies reporting both the patient burden (via medical charts) and physician perceptions of burden are lacking, and studies examining healthcare resource utilization (HCRU) and the economic burden of CS are limited. The current study reviewed medical charts of patients with CS to characterize the overall burden of CS (including symptoms, treatments, and HCRU) as well as physician perceptions of available treatments for CS and the rationale behind associated treatment decisions.

## Methods

### Study design and recruitment

This quantitative, cross-sectional study was conducted to collect disease burden data pertaining to patients with CS from qualified physician respondents. All study materials were reviewed and granted exemption by a central Institutional Review Board (IRB) prior to study execution (Advarra; Columbia, MD; https://www.advarra.com/). HCPs were recruited via a physician panel through an independent recruitment partner (Toluna) and received an appropriate honorarium for their time participating in the study.

This study was fielded between May 26 and July 27, 2021, and involved the abstraction of retrospective medical chart data from patients with a confirmed diagnosis of CS by endocrinologists. The survey included a 45–60-min web-enabled questionnaire, including a case report form (CRF) component, to capture patient medical chart data and health care practitioner (HCP)-level data in order to assess perceptions of CS disease burden, treatments, and attributes associated with treatments. Considering the rarity of CS, each HCP was required to abstract information from a minimum of 2 patient charts, and a maximum of 8 patient charts.

### Selection of study population

HCPs were able to participate in the study if they:

1. Were board-certified or board-eligible in endocrinology in the United States.
2. Had been in practice for more than 3 years and less than 35 years post residency.
3. Spent at least 25% of their professional time providing direct patient care.
4. Had treated or managed at least 40 unique patients (of any condition) in an average month.
5. Had managed (i.e., had an appointment with) at least 3 patients with CS in the past year.
6. Had access to confirmed CS patient chart(s) at the time of the study.

Each HCP who qualified to participate provided information via chart abstraction from the medical records of 2–8 patients with CS. The selected medical charts were from patients ≥ 21 years of age who had received a physician confirmed diagnosis of CS at least 3 months before the time of the study, and had received at least one therapy (surgical, radiological, or pharmacological) to treat their CS within the past 12 months. Patients who were diagnosed with adrenal or pituitary carcinomas were excluded.

### Data analysis

The data analysis was conducted in SAS 9.4 (SAS Institute Inc., Cary, NC, USA) and Q Research Software 5.6. (Q Research Software, New York, NY). After pilot interviews and throughout the fielding, quality control checks of all the case report forms were conducted to ensure that charts with logical inconsistencies were removed from the sample. Descriptive statistics (such as means, medians, and frequencies) were used to describe the study population across various patient and physician level metrics.

## Results

### Endocrinologists’ demographics and practice characteristics

Endocrinologists’ demographic information and practice characteristics are presented in Table 1. A total of 69 endocrinologists were surveyed and they provided information on 273 unique patient charts. The majority of the 69 endocrinologists surveyed (53/69, 73%) were male. The mean (± SD) time in practice was 17.3 (± 7.6) years. The majority of endocrinologists (35/69, 51%) worked in urban practices and were in private practice settings (47/69, 68%) (Table 1). The sample was almost equally distributed between physicians from the northern (26%), southern (29%), eastern (25%) and western (22%) regions of the United States. The mean (± SD) estimated number of patients with endogenous CS seen in the last 6 months was 30 (± 34.4) patients.

**Table 1:** Endocrinologist demographics and practice characteristics

Demographics	N = 69
Endocrinologist gender identity, n (%)	
Male	53 (77%)
Female	11 (16%)
Other	0 (0%)
Prefer not to say	5 (7%)
Average years in practice, mean ± SD	17.3 ± 7.6
Average number of patients with endogenous CS seen in last 6 months, mean ± SD	
Exogenous Cushing's syndrome	27.9 ± 34.6
Endogenous Cushing’s syndrome	30 ± 34.4
Average number of HCPs at primary practice setting, mean ± SD	25.4 ± 66
Primary practice location, n (%)	
Rural	5 (7%)
Suburban	27 (39%)
Urban	35 (51%)
Prefer not to say	6 (2%)
Primary practice region of US, n (%)	
North South East West Prefer not to say	18 (26%) 20 (29%) 17 (25%) 15 (22%) 5 (7%)
Practice settings, n (%)^a^	
Private practices Academic/university hospital Community hospital	47 (68%) 22 (32%) 18 (26%)
Endocrinologist from an CS COE, n (%)	
Yes No	31 (45%) 38 (55%)

^a^Endocrinologist were allowed to select multiple practice settings, if applicable

### Patient demographics

Patient demographics and clinical characteristics at the time of the survey are shown in Table 2. The majority of patients (165/273, 60%) were female with a mean (± SD) age at diagnosis of 40.2 (± 12.3) years and a mean (± SD) age at the most recent visit of 46.5 (± 13.4) years. Mean (± SD) BMI was 33.3 (± 8.3) kg/m^2^, with 50.5% of patients categorized as obese, 33.0% of patients categorized as overweight, 14.7% of patients categorized as normal or healthy weight, and 1.8% of patients categorized as underweight (Table 2). Most patients (167/273, 61%) had private or commercial health insurance. Patient demographics and clinical characteristics at disease diagnosis are shown in Table 2. A majority of patients (194/273, 79%) originally saw their primary care physician (PCP) prior to diagnosis and were diagnosed in a private practice setting (182/273, 67%). At the time of diagnosis, 46/273 patients (17%) had poor health, 107/273 patients (39%) had fair health, 68/273 patients (25%) had neutral health, 45/273 patients (16%) had good health, and 7/273 patients (3%) had excellent health, according to the responding physician.

**Table 2:** Patient demographics, clinical characteristics and therapy experience at diagnosis and time of the study

Demographics	N = 273
Female, n (%)	165 (60%)
Age (years) at diagnosis, mean ± SD	40.2 ± 12.3
Age (years) at the most recent visit, mean ± SD	46.5 ± 13.4
Race/Ethnicity, n (%)	
White or Caucasian Black or African American Hispanic or Latino Asian Biracial/multiracial Other	147 (54%) 40 (15%) 36 (13%) 22 (8%) 15 (5%) 13 (5%)
BMI (kg/m^2^) at diagnosis, mean ± SD	33.3 ± 8.3
Percent obese (≥ 30) Percent overweight (25–29.9) Percent normal or healthy weight (18.5–24.9) Percent underweight (< 18.5)	63% 28% 8% 0%
BMI (kg/m^2^) at the most recent visit, mean ± SD	31.6 ± 8.3
Percent obese (≥ 30) Percent overweight (25–29.9) Percent normal or healthy weight (18.5–24.9) Percent underweight (< 18.5)	51% 33% 15% 2%
Patient insurance type^a^, n (%)	
Physical/commercial Medicare/Medicaid Do not know/unsure Veteran’s Administration/government/military Cash-pay/none	167 (61%) 82 (30%) 21 (8%) 6 (2%) 1 (0%)
Comorbidities at diagnosis, n (%)	
Obesity Hypertension Depression Diabetes Dyslipidemia Anxiety Impaired glucose tolerance Metabolic disease Osteoporosis NASH Cardiovascular disease Bone fractures Hyperandrogenism Atherosclerosis Kidney stones Deep vein thrombosis Pulmonary embolism	109 (40%) 106 (39%) 97 (36%) 77 (28%) 71 (26%) 55 (20%) 55 (20%) 37 (14%) 26 (10%) 19 (7%) 15 (5%) 9 (3%) 7 (3%) 6 (2%) 5 (2%) 4 (1%) 2 (1%)
Other significant comorbidities (please specify)	
Sleep apnea	1 (0%)
COPD	1 (0%)
No comorbidities	51 (19%)
Patient overall health status at diagnosis, n (%)	
Poor Fair Neutral Good Excellent	46 (17%) 107 (39%) 68 (25%) 45 (16%) 7 (3%)
Most common physician types seen prior to diagnosis (i.e., > 10%), n (%)	
Primary care physician Endocrinologist Obstetrician/gynecologist	194 (79%) 83 (34%) 28 (11%)
First physician seen with CS symptoms, n (%)	
Primary care physician Endocrinologist I do not know Obstetrician/gynecologist Nephrologist Psychiatrist/psychologist Neurologist or neurosurgeon Otolaryngological surgeon (ENT) Dermatologist	155 (65%) 37 (15%) 24 (10%) 9 (4%) 4 (2%) 3 (1%) 3 (1%) 2 (1%) 1 (0%)
Diagnosing physician, n (%)	
Endocrinologist Primary care physician Otolaryngological surgeon (ENT) Neurologist or neurosurgeon Obstetrician/gynecologist Nephrologist	44 (64%) 18 (26%) 1 (1%) 1 (1%) 3 (4%) 2 (3%)
Type of therapy^b^, n (%)	N = 273
Pharmacotherapy only	87 (32%)
Monotherapy only	82 (30%)
Combination pharmacotherapy only	5 (2%)
Patients with surgery	180 (66%)
Surgery only	79 (28.9%)
Surgery and radiation	11 (4%)
Surgery and pharmacotherapy	85 (31%)
Surgery and drug monotherapy	74 (27.1%)
Surgery and drug combination	11 (4%)
Surgery and radiation and pharmacotherapy (mono or combination)	5 (2%)
Other (excluding surgery)	6 (2%)
Radiation only	2 (1%)
Radiation and pharmacotherapy (mono or combination)	4 (1%)

^a^Patients were given the option to select more than one type of insurance

^b^All treatments indicated are at the time of the study

### Treatment of endogenous Cushing’s syndrome

The patient treatment experience at the time of the study is presented in Table 2. Of the 273 patients, 79 (28.9%) underwent surgery only, 11 patients (4.0%) underwent surgery and radiation therapy, 4 patients (1.4%) underwent radiation therapy and pharmacotherapy, 5 patients (1.8%) underwent surgery, radiation therapy, and pharmacotherapy, 85 patients (31.1%) underwent surgery and pharmacotherapy, 2 patients (< 1%) underwent radiation alone and 87 patients (31.9%) underwent pharmacotherapy alone.

### Symptomatic burden of endogenous Cushing’s syndrome

**Fig. 1:**
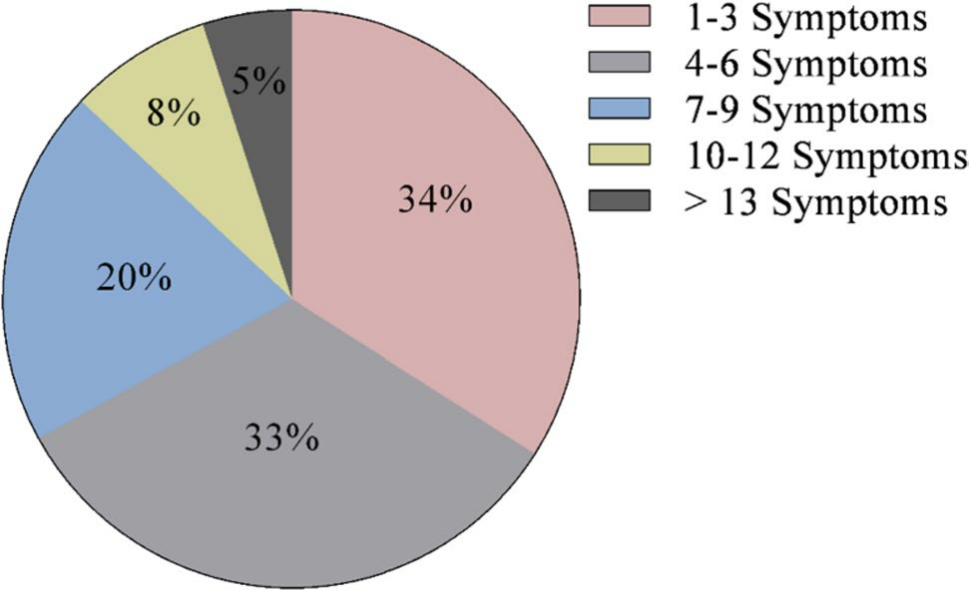
Number of CS symptoms reported at diagnosis

**Fig. 2:**
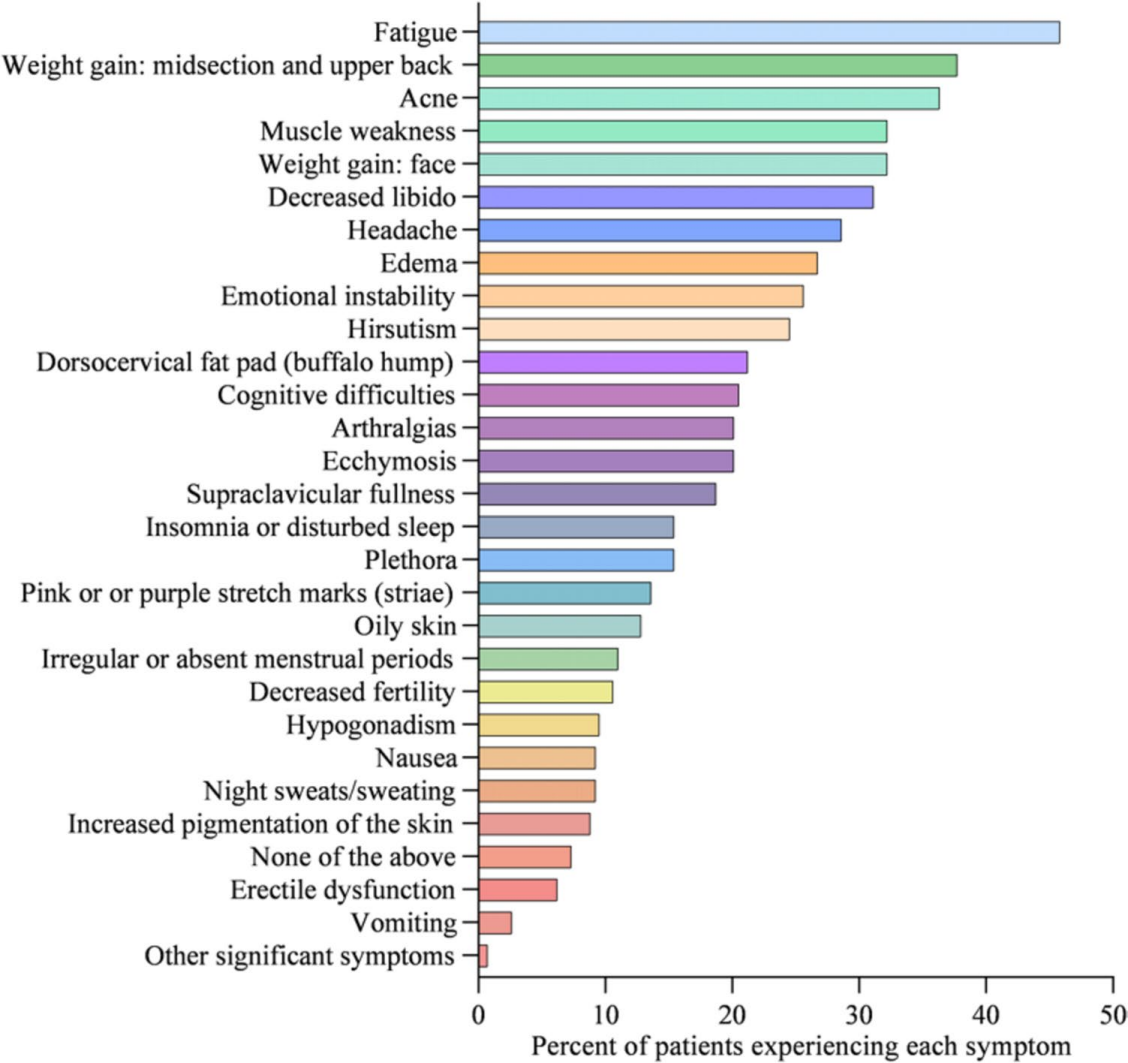
Symptoms of CS at diagnosis (N = 273)

**Fig. 3:**
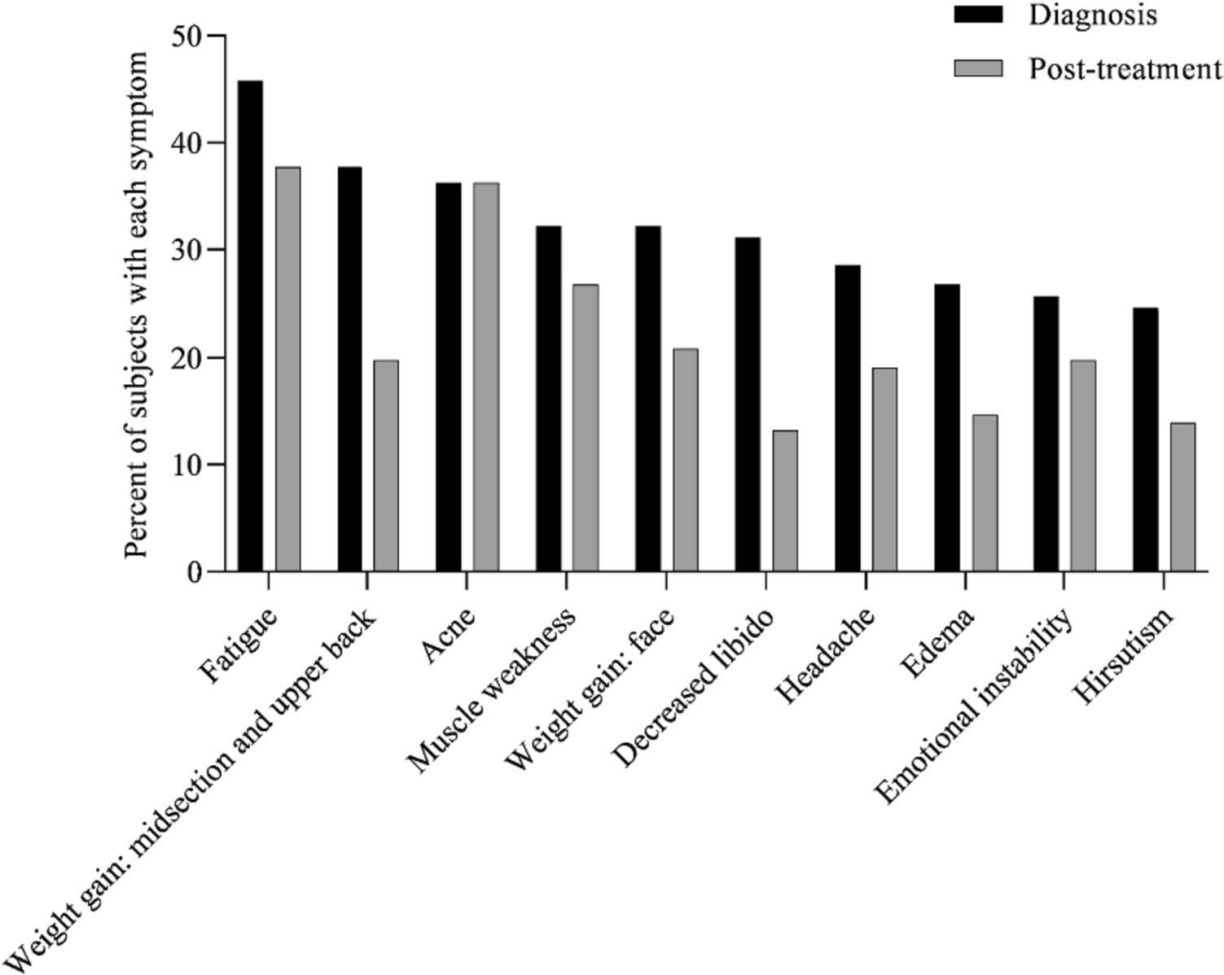
Top 10 symptoms of CS over time. Responses were restricted for Erectile Dysfunction and Irregular Menstrual Periods. Hirsutism was not restricted to females only. All denominators in the table reflect the entire patient cohort, while the metrics below are based on only the affected genders: Female Only Hirsutism: 19% of the cohort (= 52/273), 32% of the females (= 52/165), Erectile Dysfunction: 6% of the cohort (= 17/273), 16% of the males (= 17/108) and, Irregular Menstrual Period: 11% of the cohort (= 30/273), 18% of the females (= 30/165)

At diagnosis, 34% of patients presented with 1–3 symptoms, 33% of patients presented with 4–6 symptoms, 20% of patients presented with 7–9 symptoms, 8% of patients presented with 10–12 symptoms, and 5% of patients presented with > 13 symptoms (Fig. 1). Symptoms of CS at the time of diagnosis are shown in Fig. 2. The top 10 most common symptoms of CS at the time of diagnosis (Fig. 3) included fatigue, weight gain (in the midsection and upper back), acne, muscle weakness, facial weight gain (i.e., facial roundness), decreased libido, headache, edema, emotional lability, and hirsutism. Although symptoms decreased post-treatment, a large proportion of subjects still exhibited these symptoms post-treatment (Fig. 3). The most commonly reported comorbidities observed in patients with CS at the time of CS diagnosis (i.e., those affecting ≥ 20% of patients) included obesity, hypertension, depression, diabetes, dyslipidemia, anxiety, and impaired glucose tolerance (Table 2).

### Economic burden of Cushing’s syndrome

Healthcare resource utilization was assessed (Table 3). Patients required a mean (± SD) of 1 (± 1.4) hospitalization annually with a mean (± SD) length of impatient stay of 4.3 (± 3.1) days. Patients required a mean (± SD) of 0.6 (± 1.3) annual emergency room (ER) visits, and 4.3 (± 6.3) outpatient visits.

**Table 3:** Healthcare resource utilization

	Mean (days)	SD	Median (days)	N
Annual hospitalizations	1.0	1.4	1.0	273
Average length of inpatient stays	4.3	3.1	4.0	140
Annual ER visits	0.6	1.3	0.0	273
Annual outpatient visits	4.3	6.3	4.0	273

### Endocrinologists’ perceptions of disease burden

**Fig. 4:**
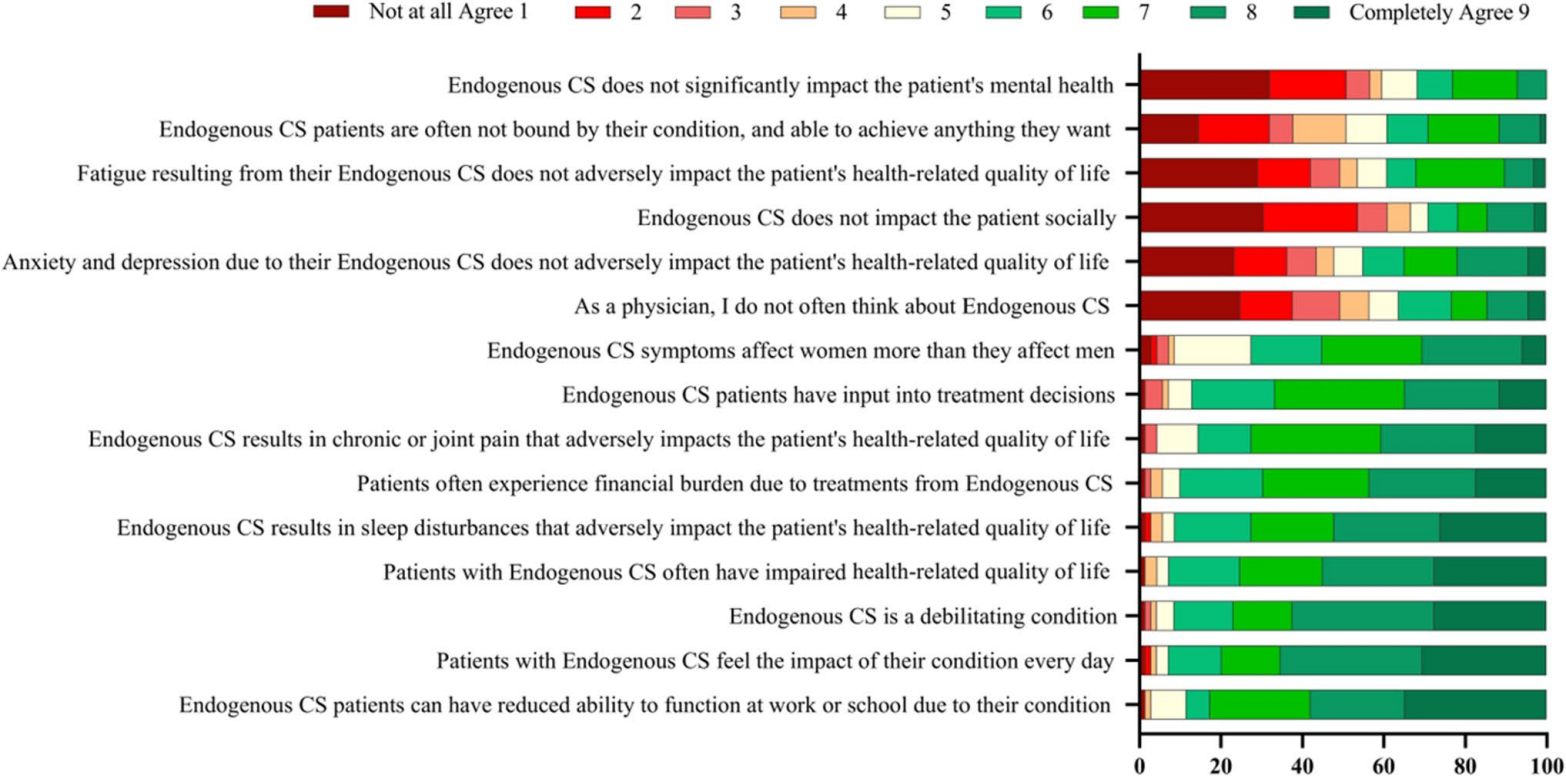
Physicians’ perceptions of CS burden and impact. On a scale of 1–9, where 1 = Not at all agree and 9 = Completely agree

Endocrinologists were asked if they agreed with a series of statements regarding their perception of CS burden and impact on a scale of 1–9, where 1 = Not at all agree and 9 = Completely agree (Fig. 4). The highest proportion of endocrinologists responded “Completely agree” with the statements “CS patients can have reduced ability to function at work or school due to their condition” (percent of endocrinologists who responded “Completely agree” = 35%), “patients with CS feel the impact of their condition every day” (30%), that “CS is a debilitating condition” (28%), “patients with CS often have impaired health-related quality of life” (28%), and “CS results in sleep disturbances that adversely impact patient’s HRQoL” (26%).

### Endocrinologists’ treatment perceptions

Endocrinologists were asked for their perceptions of the most important treatment attributes on a scale of 1 to 5, where 1 = the least important and 5 = the most important (Table 4). The two most important treatment attributes included treatments that were efficacious post-surgery (mean score = 4.0) and efficacious as a combination therapy (3.7). Endocrinologists were asked to rank satisfaction with currently available treatments for CS including surgical intervention, pharmacotherapy, and radiological or other interventions on a scale of 1–9, where 1 = Not at all satisfied and 9 = Extremely satisfied (Table 5). Overall, endocrinologists reported highest satisfaction with surgical intervention with regards to initial efficacy (mean score = 7.2), durability (6.9), safety (6.3), side effects (6.2), tolerability (6.4), and patient’s overall experience (6.9). Endocrinologists also ranked pharmacotherapy higher than radiation therapy for the treatment of CS for initial efficacy (5.9 versus 5.2), safety (5.9 versus 5.4), side effects (5.3 versus 5.2), tolerability (5.7 versus 5.5), and patient’s overall experience (5.9 versus 5.4).

**Table 4:** Top 5 highest rated treatment attributes

Treatment Attributes	Mean
(N = 69)	
Efficacious for those post-surgery	4.0
Efficacious as a combination therapy	3.7
Efficacious at decreasing visible symptoms of Endogenous Cushing's Syndrome (e.g., less hirsutism, acne, weight loss, etc.)	2.5
Efficacious at normalizing cortisol levels	2.4
Safety profile that allows for long-term utilization	2.4

^a^On a Scale of 1 to 5, where 1 = least important and 5 = most important

**Table 5:** Physicians’ satisfaction across therapeutic categories

	Mean scores ± SD		
	Surgical interventions	Pharmacotherapy	Radiological or other interventions
Initial efficacy	7.2 ± 1.6	5.9 ± 1.6	5.2 ± 2.0
Durability	6.9 ± 1.5	6.0 ± 1.3	6.0 ± 1.8
Safety	6.3 ± 1.4	5.9 ± 1.4	5.4 ± 1.7
Side effects	6.2 ± 1.4	5.3 ± 1.8	5.2 ± 1.9
Tolerability	6.4 ± 1.5	5.7 ± 1.6	5.5 ± 1.7
Patient’s overall experience	6.9 ± 1.4	5.9 ± 1.5	5.4 ± 1.9

^a^On a Scale of 1 to 9, where 1 = not at all satisfied and 9 = extremely satisfied to the question “How would you rate your satisfaction with these therapeutic categories?”

### Endocrinologists’ attitudes toward treatments and interventions

**Fig. 5:**
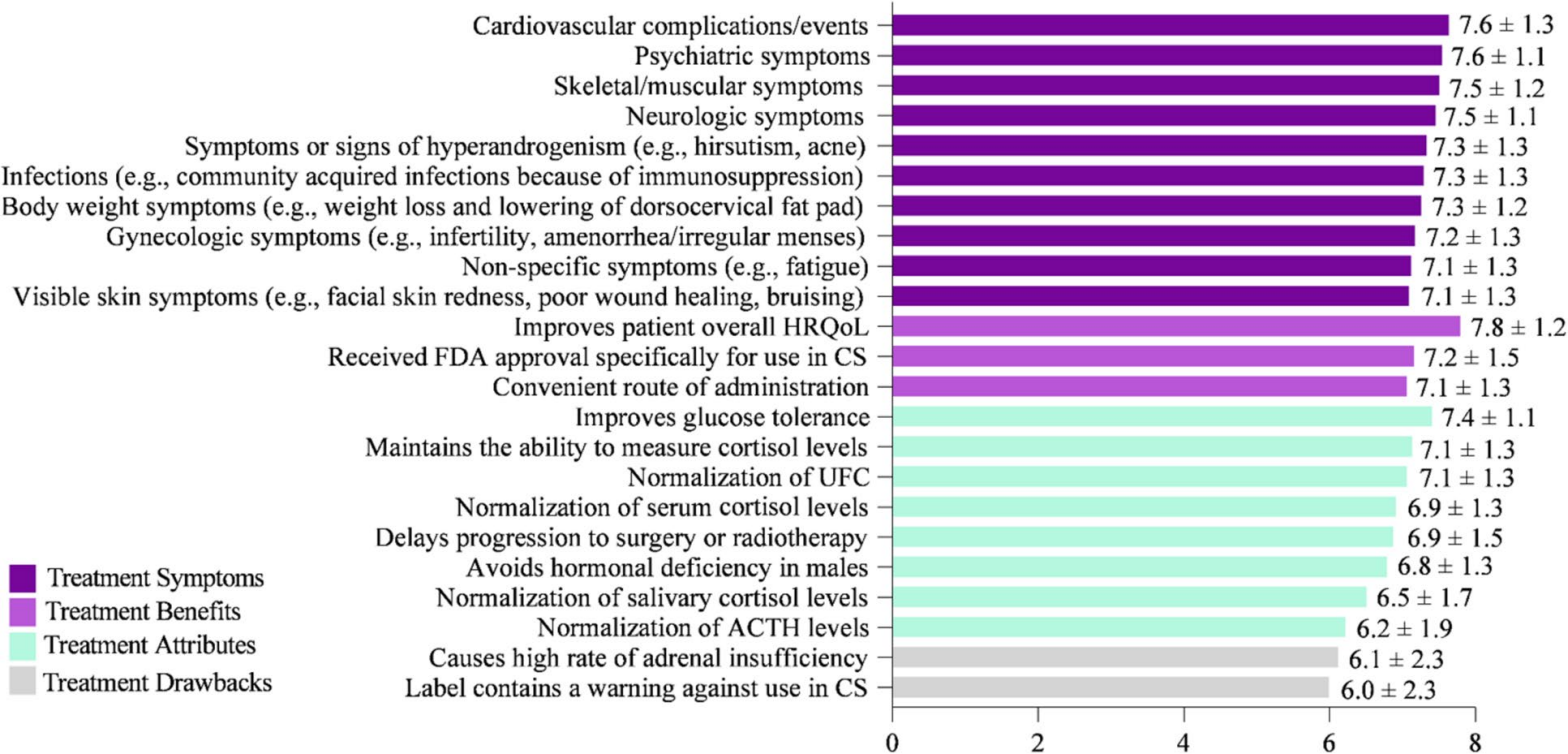
Key factors for evaluating CS treatments that influence medication selection. On a scale of 1–9, where 1 = Not at all important and 9 = Extremely important

Key factors for evaluating and selecting a CS treatment were rated on a scale of 1–9, with 1 = Not at all important and 9 = Extremely important (Fig. 5). Improving HRQoL (mean score = 7.8) was rated as the most important attribute. Similarly, improving cardiovascular complications/events (e.g., myocardial infarction, stroke, embolism) (7.6), psychiatric symptoms (e.g., depression, anxiety, mood changes) (7.6), skeletal/muscular symptoms (e.g., muscular weakness, decrease in bone mineral density, bone fractures) (7.5), and neurologic symptoms (e.g., headaches, memory, and cognitive difficulties including brain fog) (7.5) were ranked as key factors when choosing CS treatment. While factors in the survey such as “causes high rate of adrenal insufficiency” and “label contains a warning against use in CS” were ranked as less important, none of the factors listed were considered unimportant by physician respondents for choosing CS treatment.

Endocrinologists were asked if they agreed with a series of statements regarding CS treatment and intervention attitudes on a scale of 1–9, where 1 = strongly disagree and 9 = strongly agree (Table 6). The three highest scoring statements were “there is a significant clinical unmet need for patients with endogenous CS” (mean score = 6.6), “better patient support services for CS medications often leads to better patient adherence” (6.5), and “patient out of pocket cost is a significant burden for CS patients on a pharmacological therapy” (6.5). The lowest scoring statement was “patient out of pocket cost is not a significant factor when prescribing pharmacological therapy for my CS patients” (4.6).

**Table 6:** Physicians’ attitudes toward CS treatment and intervention

Attitudes	Mean score
There is a significant clinical unmet need for patients with Endogenous Cushing's Syndrome	6.6
Better patient support services for Endogenous Cushing's Syndrome medications often leads to better patient adherence	6.5
Patient out of pocket cost is a significant burden for Endogenous Cushing's Syndrome patients on a pharmacological therapy	6.5
Patients consider waiting for surgery for long periods of time to be a huge burden	6.3
Most endocrinologists recognize the signs and symptoms of Endogenous Cushing's Syndrome at first presentation	6.1
Endogenous Cushing's Syndrome medications often do not have good reimbursement support	6.0
Whether the pharmacological products had known insurance hurdles or additional paperwork is a strong factor in my prescribing decisions for my Endogenous Cushing's Syndrome patients	6.0
I think there are good options for short-term remission, however long-term remission is difficult	6.0
Treatments available today often allow patients to achieve and stay in remission	6.0
Overall, the patient support services for therapies provided by pharmaceutical companies are adequate	5.8
I am very frustrated with the side effects of current medications used for Endogenous Cushing's Syndrome	5.8
Insurance requirements (e.g., step-edits and prior authorization) for mifepristone and/or osilodrostat/pasireotide changes my treatment protocol for Endogenous Cushing's syndrome patients	5.6
A good amount of patient advocacy resources and educational materials exist for Endogenous Cushing's Syndrome patients	5.6
Current medications are excellent for management of Endogenous Cushing's Syndrome	5.5
The availability of patient support services (e.g., pharmacy team answering questions 24 × 7, nurse ambassadors, mobile application, training on dosing, etc.) for a treatment is not a factor in my prescribing decisions for my Endogenous Cushing's patients	5.4
I think Endogenous Cushing's Syndrome is not well represented in published literature	5.4
I am not comfortable prescribing pharmacological therapies without an FDA-indication specific to Endogenous Cushing's Syndrome for my patients	5.0
Patient out of pocket cost is a not a significant factor when prescribing a pharmacological therapy for my Endogenous Cushing's Syndrome patients	4.6

^a^On a scale of 1–9, where 1 = strongly disagree and 9 = strongly agree with a series of statements regarding CS treatment and intervention attitudes

## Discussion

This study provides valuable information on the physician’s perspective of unmet needs and treatment goals for patients with CS. Endocrinologists in our sample strongly agreed that patients with CS suffered from a debilitating daily condition with a high HRQoL burden. Endocrinologists also strongly agreed with the view that “there is a significant clinical unmet need for patients with endogenous CS” and ranked prescribing treatments to improve HRQoL, cardiovascular events, depression, and anxiety as key factors influencing treatment decisions. The importance providers place on the availability of post-surgery treatment options reflects the inability of many patients with CS to achieve complete post-surgical symptom resolution and suggests all symptoms in patients with CS are not currently addressed with available treatments.

Multiple treatment modalities were utilized by endocrinologists in the care of patients with CS, including surgery, pharmacotherapy, and/or radiation therapy. Improvement in HRQoL was the key treatment attribute influencing CS treatment choices, followed by the goal of reducing cardiovascular complications, and decreasing psychiatric symptoms. However, the prevalence of comorbidities after CS treatment as well as endocrinologists’ perceptions and attitudes regarding an unmet need for CS treatments and ongoing disease burden showed that few therapies are able to improve patients’ ongoing disease burden. New CS treatments are needed that have long-term efficacy, fewer side effects, and effective reimbursement.

Patients with CS have a high symptomatic disease burden at diagnosis. This study and others have demonstrated that many of these signs and symptoms (e.g., hypertension, obesity, and depression) persist even after receiving treatment aimed at normalizing cortisol levels [12–15]. Results from the present study show that many patients continue to experience fatigue, weight gain, muscle weakness, and emotional lability even after treatment, indicating an unmet need for CS treatments that can effectively manage these persistent symptoms. The persistence of symptoms after treatment for CS is likely multifactorial, and may, at least in part, be due to complications of prolonged hypercortisolism, given diagnostic and treatment delays; however, the ability to predict which patients will continue to experience persistent symptoms after treatment is challenging [14, 16, 17]. Additionally, the effects of inadequate cortisol control, symptoms due to glucocorticoid withdrawal, and side effects from medications taken to address comorbidities may contribute to persistent symptoms after treatment for CS. Although there are currently established reference values and treatment guidelines used to stratify patients, there are no current clear guidelines on management of ongoing symptoms after cortisol levels have been addressed [18]. Additionally, the present study indicated that only 32% of patients were diagnosed at the first presentation of their CS symptoms, underscoring the importance of increasing awareness of CS and its presentation among PCPs to expedite diagnosis and treatment.

The economic burden of illness from CS includes both the direct impact on HCRU, and the indirect impact on the patient due to loss of work productivity. The present study determined that the mean (± SD) annual number of hospitalization among patients with CS was 1 (± 1.4) day with an average length of inpatient stay of 4.3 days, similar in duration to the mean length of stay for all hospitalizations in the US [19]. However, the average number of outpatient visits among patients with CS was 4.3 visits per year, slightly lower than described in a recent study of patients with CS [11], but almost twice the rate of the average American, indicating a substantial direct cost burden [20]. Patients’ reduced ability to function at work or at school could limit their full economic potential, not only for themselves, but for family members and caregivers, indicating an indirect economic cost.

The degree of concordance between patients’ chart data and the perceptions of providers regarding disease symptoms is an important issue raised, but not directly addressed, by this study. Although endocrinologists agreed that there was a high HRQoL burden attributable to CS, this study did not analyze patients’ perceptions of HRQoL burden of CS. Discordance between patients’ perceptions and the perceptions of their healthcare providers, as well as the tendency of providers to perceive disease burden as less impactful or severe than is perceived by patients, has been reported in other medical conditions such as acromegaly, rheumatoid arthritis and chronic pain. The result of this is often worse medical outcomes for patients with rheumatoid arthritis or worse pain and functioning in patients with chronic pain [21–24]. Further study is necessary to analyze the concordance between the perceptions of physicians and patients with CS.

A recent cross-sectional web-enabled survey burden of illness study and a recent systemic literature review [11, 25, 26], conducted by the authors of this study, elucidated both the burden of CS as well as unmet needs in the healthcare system for patients with CS. The results of the current study corroborate the findings of both of these studies, confirming that patients experience a substantial and complex burden of cumulative CS symptoms that impacts their HRQoL. Similar to prior studies, the current results also demonstrate that although symptoms improve with treatment, some symptoms such as weight gain, pain, and anxiety persist even after treatment interventions, including surgery, pharmacotherapy, and radiation therapy. Patients with CS have previously been shown to have worse HRQoL scores compared to healthy counterparts [26], underscoring the long-term effects of CS despite treatment. This study and others have demonstrated that current therapies do not completely mitigate this HRQoL burden and indicate an unmet need among many patients with CS for additional treatments to control symptoms after cortisol level normalization.

### Study limitations

During the time in which this study was conducted, additional CS treatments could have been approved, potentially changing the treatment landscape, and thereby altering the proportion of patients that continued to have symptoms after treatment (Fig. 3) or the proportion of patients with a particular comorbidity after treatment. Physician response may have been subject to recall bias; although this may have been mitigated by the use of patient chart data the possibility that details were omitted at the time of patient visits exists. Additionally, when physicians were asked about working in a Center of Excellence, the term was not explicitly defined which may have led to varying interpretations by respondents. Due to the nature of the method used (i.e., a survey given to endocrinologists treating patients at the present time), we have limited historical chart data on the entire medical journey of each patient and all important medical events may not have been captured. For example, treatments administered to patients prior to this study (i.e., those administered by previous doctors or from a different hospital) may not be present in the patients’ charts and were not captured by our survey. Additionally, we did not capture biochemical data to make definitive statements on disease status based on patient cortisol levels. Updated guidelines on cortisol levels indicative of disease severity have recently been issued by the Pituitary Society [18], and a shift toward standardized clinical guidelines may help physicians provide timely and appropriate treatment for patients with CS. Future patient-centered research in CS should focus on identifying biomarkers associated with persistent symptoms after initial treatment, which could influence the development of guidelines for managing ongoing symptoms as current treatments are focused on cortisol management. The cohort of patients with CS included in our study is also not representative of the full spectrum of patients with CS as they were required to have received at least one pharmacological therapy to be eligible for the study. This requirement was added to our eligibility criteria as the aim of our study was to evaluate the burden of illness faced by patients with Cushing’s Syndrome, post-treatment, in the real world. Future studies evaluating concordance between patient chart data and physician perceptions of CS symptoms are also likely to be of interest. Finally, patient symptoms in this study could potentially have been masked due to the use of over-the-counter medications or other prescription treatments not fully captured in charts.

## Conclusion

Patients with CS continue to experience symptoms such as fatigue, weight gain, muscle weakness, and emotional instability even after seeking and receiving treatment, indicating an unmet need for treatments that control symptoms. Future research is needed to develop a treatment paradigm that alleviates disease burden in patients with CS and that results in long-term disease control with a favorable side effect profile.

## Data Availability

The authors confirm that all pertinent data generated or analyzed during this study are included in this manuscript or Supplementary Materials. Study participants consented to the publication of their data anonymously on an aggregate basis.
